# Caplacizumab as an add-on therapy in a 7-year-old girl with exacerbated immune-mediated thrombotic thrombocytopenic purpura, a case report and literature review

**DOI:** 10.3389/fped.2024.1448801

**Published:** 2024-08-21

**Authors:** Lara Chavaz, Laurent Cimasoni, Johanna A. Kremer Hovinga, Paul Coppo, Marc Ansari

**Affiliations:** ^1^ Division of Pediatric Oncology and Hematology, Department of Women, Child and Adolescent Medicine, Geneva University Hospital, Geneva, Switzerland; ^2^ Cansearch Research Platform for Pediatric Oncology and Hematology, Department of Pediatrics, Gynecology and Obstetrics, Faculty of Medicine, University of Geneva, Geneva, Switzerland; ^3^ Department of Hematology and Central Hematology Laboratory, Bern University Hospital, University of Bern, Bern, Switzerland; ^4^ Department for BioMedical Research, University of Bern, Bern, Switzerland; ^5^ Centre de Référence des Microangiopathies Thrombotiques, Hôpital Saint-Antoine, Sorbonne Université, Assistance Publique-Hôpitaux de Paris, Paris, France; ^6^ Service d’hématologie, Hôpital Saint-Antoine, Assistance Publique-Hôpitaux de Paris, Sorbonne-Université (AP-HP.6), Paris, France; ^7^ INSERM UMRS 1138, Centre de Recherche des Cordeliers, Paris, France

**Keywords:** immune-mediated thrombotic thrombocytopenic purpura, iTTP, caplacizumab, pediatrics, benign hematological disorders

## Abstract

The cornerstone treatment for immune-mediated thrombotic thrombocytopenic purpura (iTTP) in children is a combination of therapeutic plasma exchange (TPE), corticosteroids, and rituximab. Caplacizumab is an anti-von Willebrand factor (VWF) NANOBODY molecule approved as a frontline therapy of iTTP for adults and children aged ≥12 years. Using caplacizumab in children aged <12 years remains a gray area based on recommendations but with no marketing authorization. We report the first case of a pediatric patient with iTTP successfully treated with a caplacizumab dose adjustment of 5 mg daily based on ADAMTS13 activity. We also review all published cases of iTTP in children aged <12 years treated with caplacizumab. This is a 7-year-old girl with clinical thrombotic microangiopathy, in the absence of diarrhea and kidney injury. With a French score of 2 and a PLASMIC score of 7 (high risk), the diagnosis of TTP was suspected and later confirmed by severely low ADAMTS13 activity (<5%). Immune-mediated TTP was distinguished from the congenital one due to the presence of a functional ADAMTS13 inhibitor. Daily TPE and intravenous corticosteroids were started on day 0 (D0). Rituximab was added on D4, and due to refractoriness under daily TPE, we considered off-label administration of caplacizumab from D12. A clinical answer, with a significant increase in the platelet count, was observed within 48 h. A complete ADAMTS13 recovery was reached on D62. No major adverse events were observed during the treatment. She was discharged from the hospital over 3 months ago with a platelet count still within normal ranges. In the literature, we identified a total of four case reports describing five iTTP patients aged <12 years treated with caplacizumab, with a 100% success and tolerability rate. These published data attest to the efficacy and safety of the systematic use of caplacizumab and rituximab as frontline therapy in pediatric iTTP under 12 years of age. Therefore, prospective data are needed to support commercial authorization of caplacizumab in this subpopulation. Close monitoring of ADAMTS13 activity is particularly of interest among children to limit the number of caplacizumab injections.

## 1 Introduction

Immune-mediated thrombotic thrombocytopenic purpura (iTTP) is a specific form of thrombotic microangiopathy caused by a severe antibody-mediated deficiency of to **a** **d**isintegrin **a**nd **m**etalloproteinase with **t**hrombo**s**pondin type 1 motif, member 13 (ADAMTS13), a plasma enzyme responsible for the physiological cleavage of von Willebrand factor (VWF) (1). In the absence of ADAMTS13, VWF remains a long, multimerized compound (2, 3) that triggers the spontaneous formation of microthrombi (1). The obstructed microcirculation consumes platelets, leads to microangiopathic hemolytic anemia with schistocyte formation, and contributes to ischemic organ injuries. In the case of a suspected acute episode of thrombotic microangiopathy, the pretest likelihood of a severe ADAMTS13 deficiency can be estimated using the French (4) and PLASMIC (5) scores, both validated for adults. Definitive diagnosis is established by documentation of an ADAMTS13 activity of <10% of the normal in the presence of anti-ADAMTS13 antibodies. Close follow-up for potentially life-threatening relapses is crucial after an iTTP episode (6, 7).

Historically, iTTP treatment was based on therapeutic plasma exchange (TPE) and corticosteroids, with rituximab only given as salvage therapy. Today, early administration of rituximab has become part of the first-line therapeutic strategy (1, 8). Caplacizumab is a humanized NANOBODY molecule inhibiting the interaction between platelets and VWF by shielding the VWF A1 domain. Based on two randomized control trials, TITAN and HERCULES, caplacizumab has been approved for initial therapy of iTTP in patients aged ≥12 years and weighing ≥40 kg (9, 10). The approved dosage is a 10 mg intravenous (IV) loading bolus, followed by 10 mg daily subcutaneously (9, 10). A 5 mg daily dose was suggested for children weighing <40 kg (11). In the Phase II trial, treatment duration was set at 30 days (9) but was found to be associated with an increased risk of iTTP recurrence after discontinuation (10, 12). ADAMTS13 activity is emerging as a crucial biomarker for guiding treatment duration and preventing recurrence; however, further studies are needed to confirm the discontinuation threshold (1), which currently ranges from 10% (13) to 20% (8, 14, 15). Caplacizumab is associated with faster platelet count recovery and lower risks of recurrence, refractoriness, and mortality due to iTTP (1, 9, 10). The most commonly described adverse events are bleeding, mainly mucocutaneous, as caplacizumab induces a von Willebrand disease type 2M-like state (10).

Already rare in adulthood, iTTP is even rarer in childhood (16), where hemolytic uremic syndrome and the congenital form of TTP are more prevalent (17). Consequently, guidelines for pediatric treatment of iTTP do not exist, and treatment is often based on recommendations for adults. Although caplacizumab is not authorized under 12 years of age, some pediatric iTTP cases treated with this drug have been reported. Here, we describe a patient treated in our institution who is the first published child with iTTP receiving caplacizumab dose adjustment based on ADAMTS13 activity and review published case reports.

## 2 Case report

A 7-year-old Asian girl presented at the emergency department with spontaneous bruising and three episodes of vomiting after a week of afebrile coughing. Her medical and family histories were unremarkable. Clinical examination revealed a petechial rash with hematomas of various sizes. Laboratory studies showed low platelet count (14 G/L), hemolytic anemia (hemoglobin, 73 g/L; reticulocyte count, 164 G/L; lactate dehydrogenase, 959 U/L; haptoglobin below the detection limit; indirect bilirubin, 39.2 µmol/L; and negative direct antiglobulin test), 2% schistocytes on the peripheral blood smear, and no apparent organ involvement. Anticardiolipin IgG was 23.8 U/ml (normal, <14 U/ml), and anticardiolipin IgM, beta2-glycoprotein1 IgG, IgM, and antinuclear antibodies were negative. Complement factor C3, C4, and CH50 plasma levels were within normal range.

**Figure 1:**
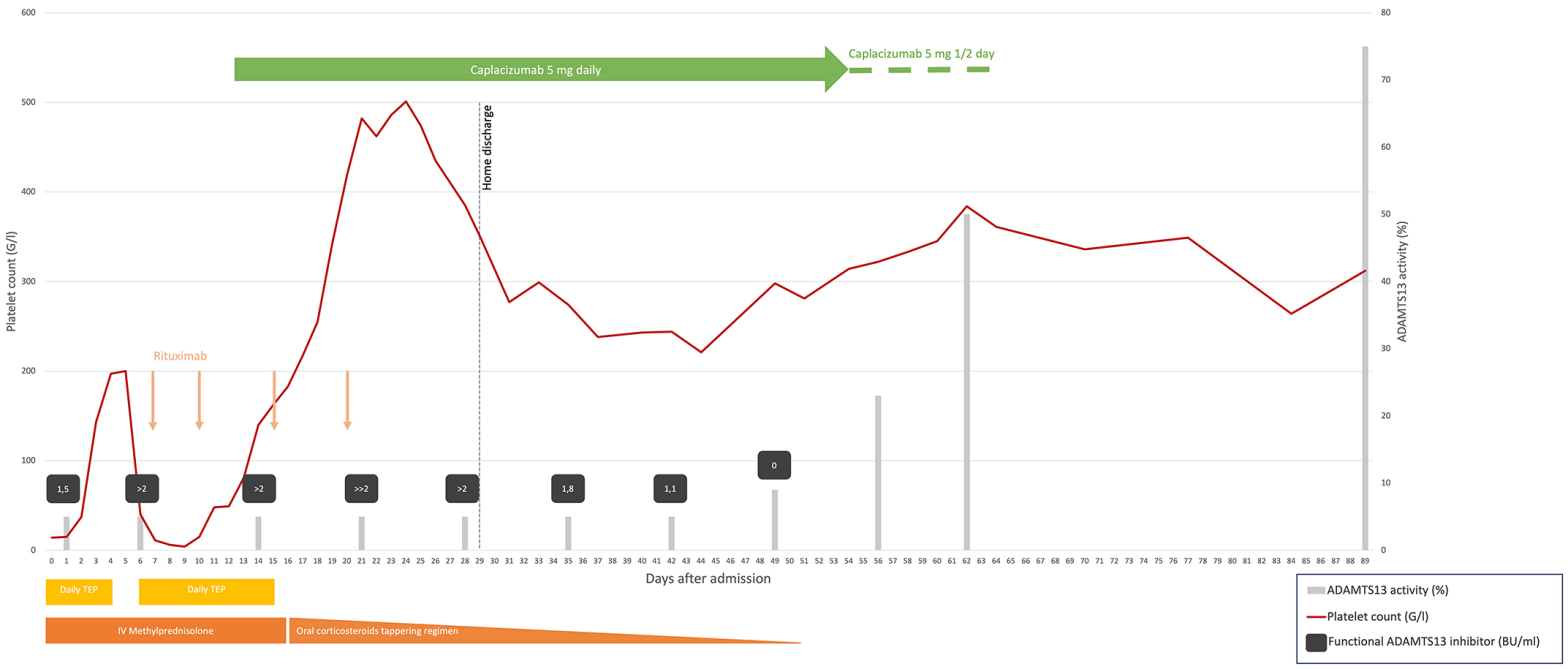
Summary of laboratory parameters and treatment administered in our case. TPE daily, days 0–4 and 6–15, temporarily increased bid days 9–12; intravenous methylprednisolone 1.5 mg/kg daily, days 0–16 temporarily increased to pulse 15 mg/kg/day, days 9–12; oral prednisone 1 mg/kg then slowly tapered, days 13–51; intravenous rituximab, 375 mg/m^2^, on days 7, 10, 15, and 20; caplacizumab 5 mg daily, intravenous day 12 and subcutaneous days 13–54, and then every second day, days 55–64. The functional ADAMTS13 inhibitor level is semiquantitative when the value is >2 BU/ml, ≫2 BU/ml, being the highest. TPE, therapeutic plasma exchange; IV, intravenous; G/L, giga/liter; BU/ml, Bethesda unit/milliliter. ADAMTS13 activity normal range, 51%–100%. Functional ADAMTS13 inhibitor normal range, <0.4 BU/ml.

The patient presented with a picture of thrombotic microangiopathy, but the absence of diarrhea and kidney injury ruled out hemolytic uremic syndrome. French score of 2 and a PLASMIC score of 7 (high risk) indicated probable TTP, soon confirmed by severely low ADAMTS13 activity (<5%), which was found to be acquired due to functional ADAMTS13 inhibitor of 1.5 BU/ml, thus ruling out congenital TTP. The patient was transferred to the pediatric intensive care unit on the day of admission (D0), receiving one packed red cell unit and fresh frozen plasma for central line placement, TPE, and intravenous (IV) methylprednisolone (1.5 mg/kg/day). Following daily TPE D0-D4, the patient showed clinical response with a platelet count of >150 G/L for 2 days, and TPE was paused. However, this was rapidly followed by an iTTP exacerbation, with the platelet count dropping to 40 G/L on D6 (Figure 1), increased hemolysis, and signs of cardiac and renal injury. TPE was restarted twice daily, with pulse methylprednisolone 15 mg/kg/day for 4 days. On D7, when the ADAMTS13 inhibitor result became available, she received the first of four doses of rituximab (375 mg/m^2^ on D7, D10, D15, and D21).

Due to refractoriness under daily TPE, we considered off-label administration of caplacizumab and contacted Sanofi's managed access program. After obtaining the parents' informed consent, our institutional review board's approval, and advice from experts in the field, caplacizumab was started on D12 with a first dose of 5 mg IV followed by daily subcutaneous administration of the same dose. Her platelet count increased to 140 G/L after 2 days and remained in the normal range thereafter (Figure 1). Hospitalization was prolonged due to central line-associated bloodstream infection (CLABSI) by methicillin-sensitive *Staphylococcus aureus* on D14. The next day, we removed all central lines and stopped TPE. After completing the rituximab course, the patient was discharged home on D29 on a tapering steroid regimen. Daily caplacizumab was continued in the outpatient unit, until D54 when, because of painful injection site reaction, it was spaced to every other day, with monitoring of trough VWF activity using the VWF:GPIbM assay and platelet counts for 10 more days.

Anti-ADAMTS13 antibody titter started decreasing by D28, with partial ADAMTS13 recovery on D56 and complete recovery on D62, respectively, 7 and 6 weeks after TPE cessation and the last rituximab infusion. The only reported side effect reported was the painful injection site reaction with local redness and petechial rash. Our patient has been at home for 3 months now with normal platelet counts. Unfortunately, we have been unable to retest the patient for the antiphospholipid antibodies after 3 months, as she moved back to her home country.

## 3 Discussion

This is the first pediatric patient successfully treated with dose adjustment of 5 mg daily and adaptation based on ADAMTS13 and VWF activities. The case underlines the risk of nosocomial CLABSI among iTTP patients dependent on a central line for their daily TPE. By reducing the number of TPE and length of stay, caplacizumab limits exposure to hospital-acquired infections. However, consideration should be given to how caplacizumab is administered to avoid real aversion to hospitalization in young children requiring chronic follow-up. Guiding treatment by measuring trough VWF activity levels allowed a reduction in the frequency of the injections.

We identified all published pediatric iTTP cases treated with caplacizumab in PubMed (“Purpura, Thrombotic, Thrombocytopenic” AND “caplacizumab” AND “Child*”) and EMBASE. Our search on 19 January 2024 found 15 and 34 results, respectively. Four case reports (18–21) described five iTTP patients aged <12 years treated with caplacizumab and are summarized in Table 1. Given the different anti-ADAMTS13 antibody and functional ADAMTS13 inhibitor tests used at the different sites, anti-ADAMTS13 antibody and functional ADAMTS13 inhibitor are not comparable and are given only as positive. Sex was evenly distributed; cutaneous, abdominal, and neurological symptoms were the most frequent clinical findings present at diagnosis. Doses of caplacizumab were either 5 mg or 10 mg daily for at least 30 days. All patients received TPE and immunosuppression with steroids, rituximab, and caplacizumab. All patients survived, and caplacizumab was well tolerated with only minor adverse events.

**Table 1:** Description of our case (in bold) and five pediatrics iTTP patients aged <12 years retrieved from the literature who were treated with caplacizumab.

Article reference	Age (years)	Sex	Platelet count on adminission (G/L)	Symptom at diagnosis	Serum creatinine elevation on admission	Troponin elevation on admission	Neurological symptoms at presentation	PLASMIC score	ADAMTS13 activity at diagnosis	Functional ADAMTS13 inhibitors	Days of TPE	Days from initiation of TPE to first dose of caplacizumab	Days of caplacizumab	Dose of caplacizumab (mg/dose)	Days to normalization of platelet count^a^	ADAMTS13 activity at discontinuation of caplacizumab	Steroid co-treatment	Rituximab co-treatment	Days of hospitalization	Days of follow-up	Bleeding complications	Recurrence after caplacizumab	Outcome
Dutt et al. (18), PMID: 33150357	3	M	9	NA	Yes	Yes	Yes	NA	<5%	Positive	16	1	27	NA	25	49,9 IU/dl	Yes	Yes	28	80	No	No	Survived
Veltroni et al. (19), PMID: 34932149	9	M	14	Abdominal pain, macrohematuria, petechial rash, hypertension	No	No	No	NA	<0.5%	Positive	6	4	30	10	2	69%	Yes	Yes	52	126	No	No	Survived
Graciaa (20)	11	M	NA	Headache, fatigue, vomiting, diarrhea	NA	NA	Yes	NA	3–20%	Positive	NA	NA	NA	NA	4	NA	Yes	Yes	NA	NA	No	No	Survived
	11	F	NA	Headache, unilateral weakness, confusion	NA	NA	Yes	NA	3–20%	Positive	NA	0	NA	NA	2	NA	Yes	Yes	NA	NA	No	No	Survived
Maitta et al. (21), PMID: 37329723	1	F	6	Bruises, fever, seizure	No	NA	Yes	NA	<5%	Positive	70	52	NA	5	6	NA	Yes	Yes	90	90	No	No	Survived
**Our case**	**7**	**F**	**14**	**Vomiting, petechial rash, bruises**	**No**	**No**	**No**	**8**	**<5%**	**Positive**	**13**	**12**	**45**	**5**	**2**	**50%**	**Yes**	**Yes**	**29**	**120**	**No**	**No**	**Survived**

All patients survived. No bleeding complications or recurrence of iTTP appeared after caplacizumab. NA, not available.

^a^ Time to normalization of platelet count is defined according to the HERCULES study protocol (10) as the time of first IV administration of caplacizumab to normalization of platelet count (i.e., ≥150 G/L with discontinuation of TPE within 5 days). Because of the different assays used to assess the presence of anti-ADAMTS13 antibodies or the function of ADAMST13 inhibitors, the anti-ADAMTS13 antibody or ADAMST13 inhibitor values are not comparable, and the result is given as positive or negative only.

These data, in line with reported experience from adult patients, support the systematic use of caplacizumab and rituximab as frontline therapy in pediatric iTTP patients. To date, there is only one retrospective clinical trial ongoing studying pediatric iTTP patients treated with caplacizumab (clinicaltrials.gov NCT05263193) with pending results (22). A randomized clinical trial on treatment with caplacizumab in children and adolescents aged <18 years or even 12 years suffering from acute iTTP would be highly appreciated, but given the low incidence rate of iTTP in children, it seems unlikely that such a study will be set up and recruit enough patients in due time. Therefore, case reports similar to the one described here or those already published are valuable in providing information and guiding physicians in treating iTTP in children.

## Data Availability

The original contributions presented in the study are included in the article/Supplementary Material; further inquiries can be directed to the corresponding author.
